# Chalcone Scaffolds, Bioprecursors of Flavonoids: Chemistry, Bioactivities, and Pharmacokinetics

**DOI:** 10.3390/molecules26237177

**Published:** 2021-11-26

**Authors:** Mithun Rudrapal, Johra Khan, Abdul Aziz Bin Dukhyil, Randa Mohammed Ibrahim Ismail Alarousy, Emmanuel Ifeanyi Attah, Tripti Sharma, Shubham Jagdish Khairnar, Atul Rupchand Bendale

**Affiliations:** 1 Department of Pharmaceutical Chemistry, Rasiklal M. Dhariwal Institute of Pharmaceutical Education & Research, Pune 411019, India; 2 Department of Medical Laboratory Sciences, College of Applied Medical Sciences, Majmaah University, Al Majmaah 11952, Saudi Arabia; j.khan@mu.edu.sa (J.K.); rn.ibrahim@mu.edu.sa (R.M.I.I.A.); 3 Health and Basic Sciences Research Center, Majmaah University, Al Majmaah 11952, Saudi Arabia; 4 Department of Microbiology and Immunology, Division of Veterinary Researches, National Research Center, Giza 12622, Egypt; 5 Department of Pharmaceutical and Medicinal Chemistry, University of Nigeria, Nsukka 410001, Nigeria; emmanuel.attah.pg00429@unn.edu.ng; 6 Department of Pharmaceutical Chemistry, School of Pharmaceutical Sciences, Siksha ‘O’ Anusandhan (Deemed to be University), Bhubaneswar 751003, India; triptisharma@soa.ac.in; 7 MET Institute of Pharmacy, Bhujbal Knowledge City, Nasik 422003, India; sunnykhairnar62@gmail.com; 8 Sandip Institute of Pharmaceutical Sciences, Nashik 422213, India; atulbendale123@gmail.com

**Keywords:** chalcone, flavonoids, biosynthesis, chemistry, bioactivities, pharmacokinetics

## Abstract

Chalcones are secondary metabolites belonging to the flavonoid (C_6_-C_3_-C_6_ system) family that are ubiquitous in edible and medicinal plants, and they are bioprecursors of plant flavonoids. Chalcones and their natural derivatives are important intermediates of the flavonoid biosynthetic pathway. Plants containing chalcones have been used in traditional medicines since antiquity. Chalcones are basically α,β-unsaturated ketones that exert great diversity in pharmacological activities such as antioxidant, anticancer, antimicrobial, antiviral, antitubercular, antiplasmodial, antileishmanial, immunosuppressive, anti-inflammatory, and so on. This review provides an insight into the chemistry, biosynthesis, and occurrence of chalcones from natural sources, particularly dietary and medicinal plants. Furthermore, the pharmacological, pharmacokinetics, and toxicological aspects of naturally occurring chalcone derivatives are also discussed herein. In view of having tremendous pharmacological potential, chalcone scaffolds/chalcone derivatives and bioflavonoids after subtle chemical modification could serve as a reliable platform for natural products-based drug discovery toward promising drug lead molecules/drug candidates.

## 1. Introduction

Chalcones (or 1,3-diaryl-2-propen-1-ones) are one of the major secondary metabolites of plants belonging to the flavonoid family. These metabolites are abundantly present in edible plants. A majority of naturally occurring chalcones is polyhydroxylated aromatic compounds, and they are considered the bioprecursors of open chain flavonoids, flavonoids, and isoflavonoids. Due to the presence of phenolic groups, chalcones have a radical quenching property, which has created interest among researchers to investigate chalcone-rich plant extracts in search for therapeutically useful compounds. The therapeutic applications of chalcones have been associated since time immemorial for the treatment of different diseases [1]. Chalconarngenin, phloretin, and its glucosidephloridzin (phloretin 2′-O-glucose) are some of the most common chalcones present in food [2].

Chalcones and their structural analogues, either natural or synthetic, are known to exhibit diverse therapeutic and pharmacological activities such as antioxidant, anti-inflammatory, antiplasmodial (antimalarial), antileishmanial, antitubercular, antimicrobial, antiviral, anticancer, modulation of P-glycoprotein (P-gp) mediated multi-drug resistance, and immunosupportive potential. Studies have revealed that compounds with a chalcone-based structure and/or chalcone template also show a profound pharmacological influence on the cardiovascular, cerebrovascular, and neurovascular systems. Some chalcones have been associated with anti-peptic ulcer and antihypertensive activities. Some other activities have been also reported as anti-spasmodic, tranquilizing, analgesic, sedative, anti-thrombic, vasodilatory, estrogenic, anesthetic, anti-coagulating, anti-convulsant, and diuretic activities. Moreover, chalcones are considered as important pharmacophores of various bioactive natural products and therefore display a variety of biological potential. Representative examples of naturally occurring bioactive chalcones are cardamonin, a hydroxychalcone isolated from a Zingiberous plant species, which possesses antimutagenic, vasorelaxant, and anti-inflammatory properties, and xanthohumol, the principal prenylated flavonoid of the hop plant, which is characterized as a broad-spectrum cancer chemopreventing agent in vitro [3,4].

This review aims to discuss detailed aspects of naturally occurring chalcones including their biosynthesis, chemistry, and spectrum of bioactivities. In addition, this review also highlights the bioavailability issues associated with natural chalcones, along with pharmacokinetics and toxicities. All the relevant databases available in electronic search engines such as Web of Science, ScienceDirect, Pubmed, and Scopus were explored to collect relevant information for the terms such as chalcones, natural and dietary chalcones, chalcone derivatives, pharmacological activities, and the bioavailability or pharmacokinetics of naturally occurring chalcones.

## 2. Chalcone: Structure, Nomenclature, and Chemistry

Chalcone is a vital intermediate substance in the biosynthetic pathway of flavonoids. The term chalcone was coined by Kostanecki and Tomar who first demonstrated chalcone as banzalacetophenone or benzylidene acetophenone [5]. In recent years, the chemistry and synthesis of chalcone-based bioactive molecules have become an interesting area of research in the field of medicinal chemistry and drug discovery for their potential as a good structural synthon with wide molecular diversity (natural as well as synthetic) and having an array of biodynamic or pharmacological activities. 

### 2.1. Chemical Structure 

**Figure 1:**
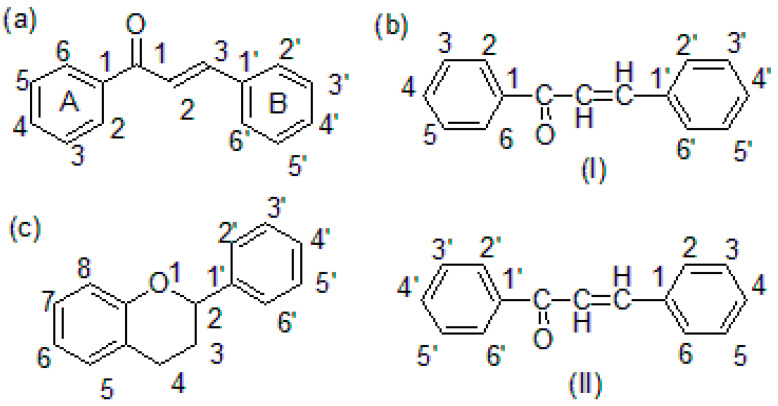
(**a**) Chalcone structure, (**b**) chalcone nomenclature (I and II), and (**c**) flavonoid skeleton.

Chalcones are α,β-unsaturated ketones containing a reactive ketoethylenic group i.e., –CO-CH=CH-. These compounds are also known as benzalacetophenone or benzylidene acetophenone. Chemically, chalcones are 1,3-diaryl-2-propen-1-one, in which two aromatic rings are linked by an aliphatic three-carbon α,β-unsaturated carbonyl system (Figure 1a). Chalcones possess conjugated double bonds and a completely delocalized π-electron system on both benzene rings. They constitute the skeleton of open-chain flavonoids in which the three-carbon aliphatic system is used as an adjunct between two aromatic rings A and B [6]. Chalcones are small, low molecular weight (in the range of 300–600 g/mol), non-chiral molecules with relatively high lipophilicity (Log P ≈ 5–7). As a result of the presence of the chromophore -CO-CH=CH-, chalcones are colored compounds. Chalcones may exist as either *cis* (E, 1) or *trans* (Z, 2) isomeric forms. The *trans* form is thermodynamically more stable than the *cis* form [6].

### 2.2. Nomenclature

Chalcone or chalconoid is an enone and an aromatic ketone, which forms the central core for several important biological compounds. Benzylideneacetophenone is the parent member of the chalcone series. The alternative names given to chalcone are benzalacetophenone, phenyl styryl ketone γ-oxo-α,γ-diphenyl-α-propylene, α-phenyl-β-benzoylethylene, and β-phenylacrylophenone. Different methods of nomenclatures for chalcone are available. The following two nomenclatures have been adopted by the “Chemical Abstracts” published by American Chemical Society (I) and the British Chemical Abstract and Journal of Chemical Society (II) (Figure 1b). The IUPAC name of chalcone is 1,3-diphenyl-2-propen-1-one [6].

### 2.3. Occurrence of Chalcones

Chalcones are secondary plant metabolites, belonging to the flavonoid family that are abundantly present in edible plants, particularly fruits and vegetables. Therefore, chalcones belong to an important class of plant flavonoids (C_6_-C_3_-C_6_ system) (Figure 1c). Chalcones and their derivatives are important intermediates of the flavonoid biosynthetic pathway. Flavonoids are an important group of naturally occurring bioactive compounds. The majority of naturally occurring chalcones are polyhydroxylated aromatic compounds abundantly found in fruits, grains, legumes, vegetables, and beverages such as tea, coffee, red wine, beer, etc. The medicinal benefits of polyhydroxylated chalcones are mainly attributed due to their free radical scavenging activity (antioxidant property), which in turn mitigates oxidative stress-induced tissue damage associated with some chronic disorders such as cardiovascular diseases, inflammatory diseases and neurological disorders, and certain infectious diseases [7,8,9].

## 3. Biosynthesis of Chalcones

**Figure 2:**
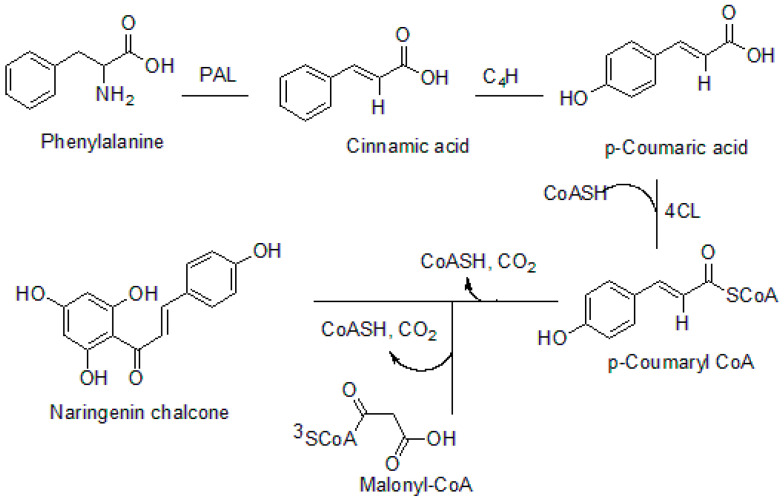
Biosynthesis of chalcone. PAL: phenylalanine ammonia-lyase, C4H: cinnamate 4-hydroxylase, 4CL: 4-coumarate-CoA ligase.

Chalcone is one of the precursors in the biosynthesis of flavonoids, isoflavonoids, anthocyanidins, proanthocyanidins, and other polyphenolic compounds [7]. Chalcone synthase (CHS) is the major enzyme that plays a vital role in the biosynthesis of chalcones [8,9]. The effectiveness of chalcone synthase (CHS) as an enzyme for chalcone formation is brought about by the presence of two active sites in the enzyme. One of the active sites referred to as the upper domain consists of four amino acids. The second active site referred as the lower domain is also essential for chalcone formation [7]. Phenylalanine is the major precursor for chalcones biosynthesis (phenylalanine is formed from chorismate as a precursor). *p*-Coumaroyl CoA and malonyl CoA are other important biomolecules required for the formation of chalcones. However, *p*-coumaroyl CoA is formed from phenylalanine [9]. Phenylalanine undergoes deamination at the aliphatic chain to form cinnamic acid. This is catalyzed by phenylalanine ammonia-lyase (PAL), which is followed by hydroxylation at the *para* position of the phenylalanine aromatic ring mediated by cinnamate-4-hydroxylase to form *p*-coumaric acid. Succinyl-CoA substitution of the hydroxyl group occurs at the aliphatic carboxyl group of the *p*-coumaric acid to yield *p*-coumaroyl CoA by the enzyme 4-coumaroyl-coenzyme A ligase. CHS catalyzes the condensation of three molecules of malonyl CoA and *p*-coumaroyl CoA (one molecule) successively. The process also involves the decarboxylation, cyclization, and aromatization of malonyl CoA, which is mediated by the four amino acids (Asn 336, His 303, Phe 215, and Cys 164) present in the active site of CHS [9]. The biosynthesis of chalcones is depicted in Figure 2.

**Figure 3:**
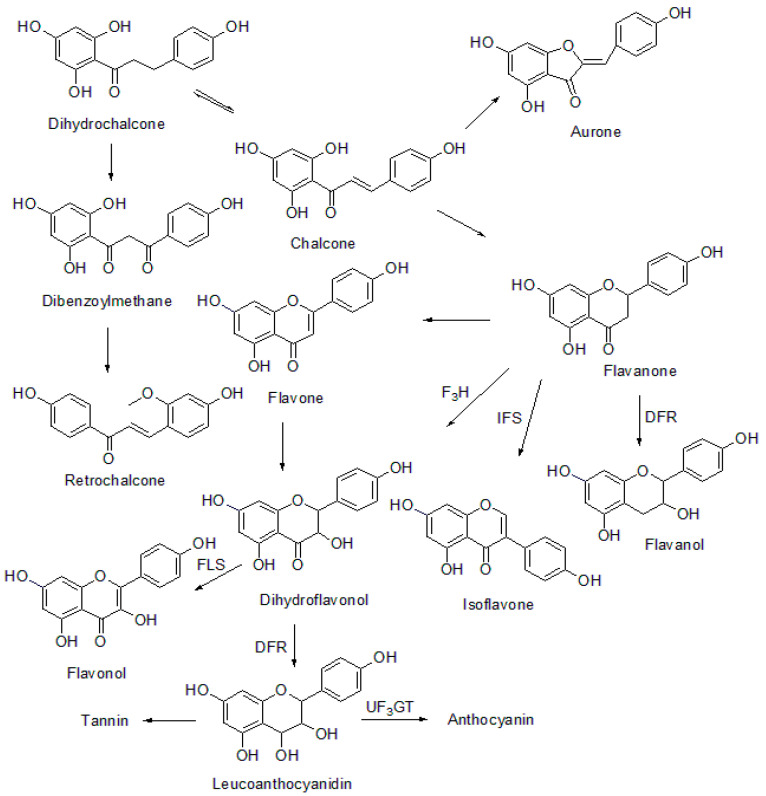
Biosynthesis of chalcone precursors. DFR: dihydroflavonol-4-reductase, IFS: isoflavonone synthase, F3H: flavanone-3-hydroxylase, FLS: flavonol synthase, UF3GT: UDP-glucose flavonoid-3-O-glucosyltransferase.

The chalcone formed is a biosynthetic precursor for various polyphenolic classes of natural products such as flavanones, flavonols, flavanols, dihydroflavonols, isoflavones, flavones, isoflavonoids, aurone, and anthocyanidins [4]. The biosynthesis of various chalcone bioprecursors is represented in Figure 3.

**Figure 4:**
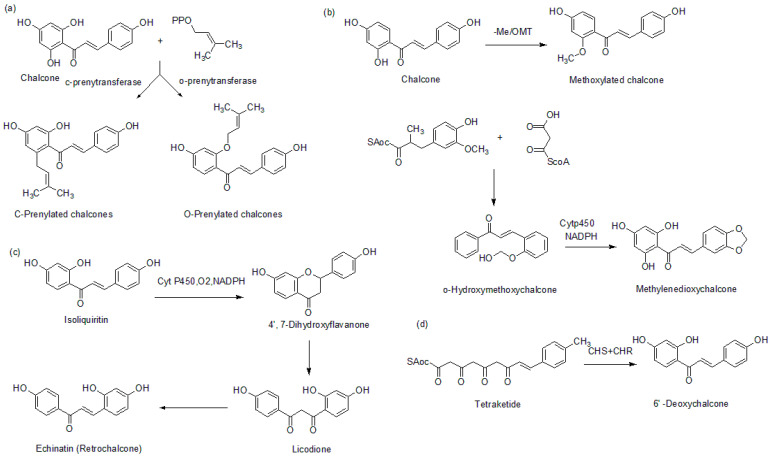
Biosynthesis of (**a**) prenylated chalcones, (**b**) methoxychalcone and methylenedioxychalcone, (**c**) retro chalcones, and (**d**) dedoxychalcones.

The formation of prenylated chalcones has been reported to be mediated by prenyltransferase, which plays a significant role in transferring prenyl units to an acceptor molecule from an isoprenyl source, which is usually dimethylallyl pyrophosphate (DMAPP) (Figure 4a) [10].

In the formation of methoxylated chalcones, methylation takes place through a catalytic mediation of S-adenosyl-L-methionine-dependent-*O*-methyltransferase (OMTs) [11]. It mediates the transfer of a methyl group from a donor (*S*-adenosyl-L-methionine) to an acceptor molecule. Methylenedioxy chalcone is generated through the formation of methylenedioxy bridges and catalyzed by cytochrome P450-dependent enzymes alongside NADPH, which acts as a cofactor (Figure 4b) [12,13]. Retro chalcones have been reported to be formed by the inversion of α, β-unsaturated ketone during the biosynthesis of 6′-deoxychalconeisoliquiritigenin (Figure 4c) [14]. It has been reported that the presence of CHS and a polyketide reductase (CHR) as the active enzymes in a biosynthetic process generates 6′-deoxychalcones (Figure 4d) [13].

During chalcone biosynthesis, the linkage of a sugar molecule catalyzed by the enzyme uridine diphosphate glycosyltransferase yields glycosylated chalcones. In this case, a nucleophilic substitution reaction is used to transfer the sugar molecule from a donor molecule (UDP-glycoside) to an acceptor molecule [15,16].

## 4. Naturally Occurring Chalcones

Chalcones occurring in nature have plants as their major source. They are usually found either in medicinal plants or in dietary plants. In nature, chalcones can be found as chalcone derivatives and flavonoids [17]. Chalcone derivatives of medicinal importance can be chemically synthesized in the laboratory by chemical modification of the parent chalcone scaffolds with a diverse range of structural substitutions [18]. 

### 4.1. Chalcones from Medicinal and Dietary Plants 

Several chalcones with proven therapeutic activities have been isolated from various medicinal and potential medicinal plants. Star et al. (1978) carried out the isolation of *Pityrogramma triangularis* [19] exudate, which yielded a chalcone, 2,6-dihydroxy-4-methoxy-3-methyl chalcone, which was reported as a new compound. Isoliquiritigenin, isoliquiritin, neoisoliquiritin [20], licochalcone A, licochalcone B [21], echinatin [22], licuroside [20], and neolicurosid [23] have earlier been isolated from liquorice (*Glycyrrhiza glabra*), which is a medicinal plant having therapeutic uses against many human diseases [20]. Two dihydrochalcones, 2,6-dihydroxy-4-methoxy-3,5-dimethyl dihydrochalcone and 4,4,6-trimethyl-2-(3-phenyl propionyl)-cyclohexane-1,3,5-trione from *Myrica gale* have been reported by Uyar et al. (1978) [24]. *Crotalaria prostrata*, an Indian medicinal plant, has been reported to yield crotaoprostrin on isolation [25]. *Psoralea corylifolia*, a known traditional medicine for Indians and Chinese, has also yielded bavachromanol, a novel natural chalcone [26]. Dihydrochalcone, dihydroisocordon, and flemistrictin B have been isolated from *Lonchocarpus xuul* root extract [27]. In a comprehensive review by Wang et al. (2020), about 42 chalcones isolated from licorice have been reported [28]. Brackenin is a dimeric dihydrochalcone isolated from *Brackenridgea zanguebarica* belonging to the *Ochnaceae* plant family [29]. Six chalcones have been isolated from *Angelica keiskei* extracts by column chromatography [30]. Alongside a flavonoid mixtecacin, oaxacin had been isolated from *Tephrosia woodii* [31] and epoxychalcone has been isolated from *Tephrosia carrollii* [32]. Furthermore, 3,4-dimethoxy chalcone and 3,4-dihyroxy-3′,4,4′-trimethoxy chalcone have been isolated from *Arrabidaea brachypoda* flowers [33]. Three chalcones, flavokawain B, pinostrobn, and pashanone have been also been reported from seeds of *Periscariala pathifolia* through chromatographic separations [34]. Four chalcones, 5,7-dihydroxy-4-phenyl-8-(3-phenyl-trans-acry-loyl)-3,4-dihydro-1-benzopyran-2-one, 2′-hydroxy-4′,6′dimethoxychalcone, 2′,4′-dihydroxy-6′-methoxy-3′,5′-dimethylchalcone, and 2′,4′-dihydroxy-6′-methoxy-3′-methylchalcone (along with three new chalcone derivatives, parasiticin A, parasiticin B, and parasiticin C) have been isolated from the fern *Cyclosorus parasiticus* [35]. Chalcones has been identified as a complex mixture of multicomponents in *Helichrysum rugulosum* [36]. *Glycyrrhizae radix* has been identified as a source of licuraside and isoliquiritin, which are nothing but chalcone derivatives [37]. Mallotophilippens C, D, and E are chalcone derivatives that have been isolated from the fruits of *Malotus philippinensis* [38]. About five chalcones have been isolated from *Artocarpus nobilis* 2′,4′,4-trihydroxy-3′-geranylchalcone, 2′,3,4,4′-tetrahydroxy-3′-geranylchalcone, 2′,4′,4-trihydroxy-3′-[′2-hydroxy-7-methyl-3-methylene-6-oetaenyl] chalcone, 2′,4′,4-trihydroxy-3′-[6-hydroxy-3,7-dimethyl-′2(E),7-oetadienyl] chalcone, and 2′3,4,4′-tetrahydroxy-3′-[6-hydroxy-3,7-dimethyl-2(E),7-octadienyl] chalcone [39]. 2-hydroxy-4′, 6′-dibenzyloxy chalcone, and 4′, 6′, 8′-trihydroxy chalcones have been isolated from *Helichrysum gymnocomum* [40]. Other compounds that have been isolated from *Bidens tripartitus* are 2′-hydroxy-4,4′-dimethoxychalcone [41]. Ponganones I and II have been identified as chalcone constituents of *Pongamia pinnata* [42]. 2′,4′-dihydroxy-3′-methoxychalcone and 2′,4′-dihydroxychalcone have been reported as constituents of *Zuccagnia punctata* [43]. 2′,4′-dihydroxy-3′,5′-dimethyl-6′-methoxychalcone has been reported from *Dalea versicolor* [44]. Cycloaltilisin 6, a dimeric dihydrochalcone, has been identified as a constituent of the bud cover of *Artocarpus altilis* [45]. Stipalen, which is a diprenylated chalcone, has been reported as a constituent of *Dalbergia stipulacea* root [46]. 3,3′dihyroxy chalcone has been isolated from *primula macrophylla* [47]. Even though flemistrictin A has been previously isolated from *Tephrosia spinosa*, two chalcones later, spinochalcones A and B, have been identified [48]. 4′-O-α-D-(2″-p-coumaroyl)glucopyranosyl-4,2′,3′-trihydroxychalcone, 4′-O-α-D-(2″-p-coumaroyl-6″-acetyl)glucopyranosyl-4,2′,3′-trihydroxychalcone, and 3′-(3-methyl-2-butenyl)-4′-O-â-D-glucopyranosyl-4,2′-dihydroxychalcone, and 4′-O-α-D-(2″-acetyl-6″-cinnamoyl)glucopyranosyl-4,2′,3′-trihydroxychalcone, which are chalcone glycosides, have been isolated from *Maclura tinctoria* [49]. *Calythropsis aurea* crude extract yielded calythropsin and dihydrocalythropsin on isolation [50]. Cedreprenone, 2′-methoxy helikrausic chalcone, cedrediprenone, 5,7-dimethylpinocembrine, flavokawin B, and uvangoletin have been isolated from the fruits and seeds of *Cedrelopis grevei* [51]. *Anneslea fragrans var. lanceolata* yielded eight dihydrochalcones, davidigenin-2′-O-(6″-O-4‴-hydroxybenzoyl)-β-glucoside, davidigenin-2′-O-(2″-O-4‴-hydroxybenzoyL)-β-glucoside, davidigen-2′-O-(3″-O-4‴-hydroxybenzoyl)-β-glucoside, davidigenin-2′-O-(6″-O-syringoyl)-β-glucopyranoside, 1-O-3,4-dimethoxy-5-hydroxyphenyl-6-O-(3,5-di-O-methylgalloyl)-β-gluco-pyranoside, davidioside, 4′-O-methyldavidioside, and davidigenin on isolation by chromatography [52]. Another two dihydrochalcones, 2′,4,4′,6′-tetrahydroxy-5-(*E*-3, 7-dimethylocta-2,6-dienyl)-3-(3-methylbut-2-enyl)dihydrochalcone, and 2′,4,4′,6′-tetrahydroxy-3,5-di(3-methylbut-2-enyl)dihydrochalcone have also been isolated from the aerial parts of *boronia inconspicua* [53]. Hostmanin A, B, C, and D, 2′,6′-dihydroxy-4′-methoxy, linderatone, aductine E, and (-)-methyl linderatin are all dihydrochalcones isolated from *piper hostmannianum var. berbicense* [54]. 2′,4′-dihydroxy-6′-methoxy-3′,5′-dimethylchalcone has been extracted from the dried flower, *Cleistocalyx operculatus* [55]. The roots of *lonchocarpus sericeus* yielded derricin and lonchocarpin on isolation of its hexane extract [56]. In addition to pedicin, two novel condensed chalcones, fissistin and isofissistin, have been isolated from the ethyl acetate extract of *Fissistigma lanuginosum* [57]. Four dihydrochalcones, 10′,6′-diacetoxy-4,4′-dimethoxydihydrochalcone, 4,2′,6′-trihydroxy-4′-methoxy dihydrochalcone, 2′,6′-dihydroxy-4′-methoxydihydrochalcone, and chalcone 2′,4′-diacetoxy chalcone have been reported from the leaves of *Carthamus arborescens* [58]. *Syzygium samarangense* has been identified as a source of stercurensin, cardamonin, and 4′, 6′-dihydroxy-2′-methoxy-3′,5′-dimethyl chalcone [59]. Litseaone A and B have been isolated from the stem bark of *Litsea rubescens* and *Litsea pedunculata* [60]. Cyclohexanyl chalcone and panduratin have been found to be a constituent of *Boesenbergia rotunda* [61]. *Crotalaria trifoliastrum* yielded munchiwarin, which has a 2,2,6-tri-isoprenyl-cyclohex-5-ene-1,3-dione skeleton [62]. *Glycyrrhiza inflate* has been reported to contain kanzonol, licochalcone A, D, and G, licoagrochalcone A, isoliquiritigenin, 5-prenyl butein, and echinantin [63]. Isoliquiritigenin, syzalterin, L-farrenol, and L-liquiritigenin have been isolated from *Pancratium maritium* [64]. Xanthohumol has been reported from *Humulus lupulus* [65]. Glabridin, licochalcone A, isoliquiritigenin, glycycoumarin, glycerin, glycerol, and liquiritigenin have been reported from *Glycyrrhiza uralensis* [66]. α-Hydroxy dihydrochalcones together with the novel isoflavanone, norisojamicin have been isolated from the *Millettiaus aramensis* stem bark [67]. A prenylated chalcone, 2′,4′-dihydroxy-5- prenylchalcone has been isolated from the aerial parts of *Lonchocarpus cultratus* [68]. *Ulvaria dulcis* has yielded 2′,3′-dihydroxy- 4′,6′-dimethoxychalcone [69]. Pashanone, pinostrobin, and flavokawain chalcones have been identified as constituents of *persicaria lapathifolia* seeds [34]. *p*-Hydroxy benzaldehyde, dorsmanin A, 4,2,4-trihydroxy-3-prenylchalcone, and 4,2,4-trihydroxychalcone have been isolated from *Dorstenia zenkeri* [70]. Ethanolic extract of *Haematoxylum campechia num.* L. has been reported to contain two chalcones, sappanchalcone and 3-deoxy sappanchalcone [71]. Some newer chalcones such as (-)-hydroxypanduratin A, a cyclohexenyl chalcone derivative, dihyro-5,6-dehydro kawain, pinocembrin, panduratin A, pinostrobin, and sakuranetin have been investigated by Tuchinda et al. (2002) [72]. Perez- Gutierrez et al. isolated six flavonoids from the bark of *Eysenhardtia polystachiya* with 2′,4′-dihydroxychalcone-6′-O-β-d-glucopyranoside, α,4,4′-trihydroxydihydrochalcone-2′-O-β-d-glucopyranoside, α,3,2′,4′-tetrahydroxy-4-methoxy-dihydrochalcone, and 3′-C-β-glucopyranosy-6′-O-β-d-glucopyranoside bearing the chalcone moiety [73]. Artoindonesianin J, a prenylated chalcone has been isolated from the root bark of *Artocarpus bracteate* [67]. More recently, Nchiozem-Ngnitedem et al. have isolated eight known chalcones alongside four new steroidal sapogenins and a conjugated chalcone–stilbene [74]. 

Chalcone has been identified as one of the vital constituents of some edible plants [75]. In general, the phenolic chalcones present in edible plants play an essential role in maintaining good health condition for humans, with their basic function ranging from being good antioxidants to antimicrobial activities, among others [76]. Chalcones have been identified, isolated, and characterized from edible plants in many research works. Phloretin-3′,5′-di-C-β-glucopyranoside, a dihydrochalcone, and chalconaringenin have been identified from *Solanaceae specie* of tomatoes [77]. Iijima et al. (2008) reported the presence of eriodictyol chalcone in tomatoes (*Solanum lycopersicum*). They also reported narigenin chalcones [78]. Slimestad and Verheul (2011) reported the presence of chalconarigenin from fresh cherry tomatoes [79]. Two hydroxylated polymethoxychalcones have been isolated from sweet orange (*citrus sinensis*) peel [80]. 2′-hydroxy-3,4,4′,5′,6′-pentaethoxychalcone and 2′-hydroxy-3,4,3′,4′,5′,6′-pentaethoxychalcone, which are C-methylated chalcones, have been isolated from the edible *syzygium samarangense* methanolic extract [59]. Apple fruit (*Malus domestica)* has been reported to possess phloridzin, seboldin, and trilobatin [81]. *Angelica keiski* (Ashitaba), which is vital as a food supplement, constitutes some chalcone compounds. Nine chalcones have been isolated from this plant alongside four coumarins in different research; 4-hydroxy derricin and xantholangelol were isolated from the ethanolic extract [82]. *Glycyrrhiza glabra*, a licorice species, is a vital constituent of candies, snacks, beverages, and sweets [28]. Many compounds including isoliquiritin apioside [83], lucuraside [84], butein-4-O-β-D-glucopyranoside [28], neoisoliquiritin [28], licochalcone C, licoagrochalcone B, licoagrochalcone C, licoagrochalcone D, kanzonol Y [85], echinatin, licochalcone B, morachalcone A, 2,3′,4,4′-tetrahydroxy-3,5′-diprenyl chalcone, 3,3′,4,4′-tetrahydroxy-2′-methoxy-5-prenylchalcone, paratocarpin B, 2,3′,4,4′,α-pentahydroxy-3,5′-diprenyl-dihydrochalcone, 2,3′,4,4′,α-pentahydroxy-3-prenyl-dihydrochalcone [86], kanzonol B, 4-hydroxylonchocarpin [87], licochalcone G [88], 3,4,3′,4′-tetrahydroxy-2-methoxychalcone [89], glypallichalcone [90], paratocarpin A and B [91], glycybridin A, B, and C have been isolated from this plant [92]. Trankoontivakorn et al. (2001) isolated six chalcones, panduratin A, pinostrobin, cardamonin, pinocembrin, 4-hydroxy panduratin A, and 2′,4′,6′-triydroxychalcone from finger root rhizomes (*boesenbergia pandurate*) [93].

### 4.2. Bioactivities of Naturally Occurring Chalcones 

**Figure 5:**
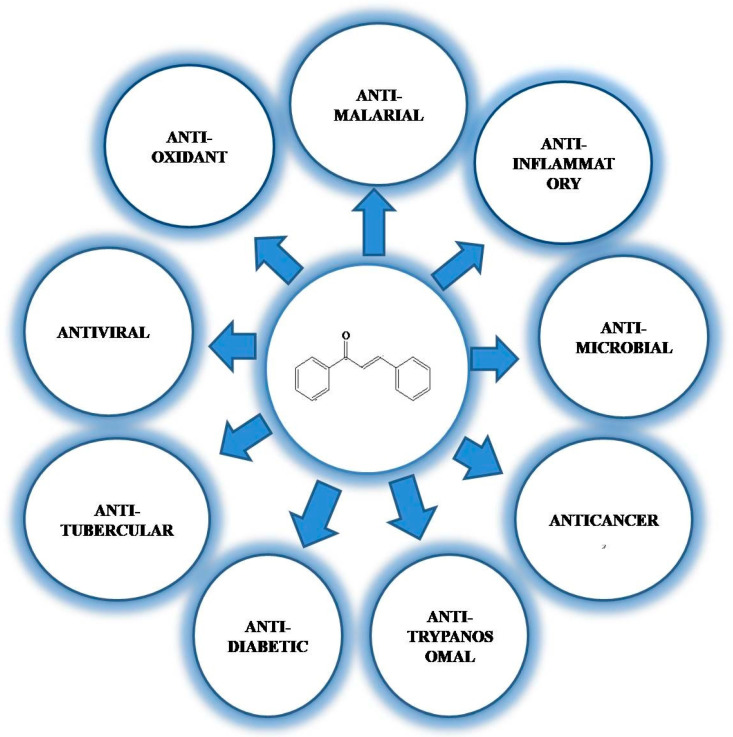
Diverse biological activities of chalcones.

Generally, chalcones exhibit a wide range of biological activities: antioxidant, antimalarial, anti-inflammatory, antimicrobial, antiosteoporosis, antiplasmodial, anticancer, antifungal, antihyperglycemic, and many others (Figure 5) [75]. Specifically, chalcones from medicinal plants exhibit these biological activities, and as a consequence, plants containing chalcones are used as therapeutic agents in various diseases. Many plants containing chalcones have shown inhibition against cancer growth. Licochalcone A, xanthohumol, 4-hydroxyderricin, butein, phloretin, garcinol, flavokawain A, B, and C, broussochalcone, dimethyl amino chalcones, cardamonin, and 2′-hydroxy-2,3,4′,6′-tetramethoxy chalcone have been reported to exhibit anticancer activity against various cancer cells [94,95,96,97]. 

The antimalarial and antileishmanial activities of some chalcones, for example, Licochalcone A, have also been investigated [98]. Chalcones from the plants *Mallotus hilippinensis* and *Maclura tinctoria* have been shown to possess antifungal activity [99]. Xanthoangelol and 4-hydroxyderricin, constituents of ashitaba, have been reported to possess a considerable extent of hyperglycemic activity [100]. Protein tyrosin phosphatase IB (PTBIB) plays a significant role in the regulation of hyperglycemia [101]. Some chalcone derivatives from medicinal plants are essential PTPIB inhibitors [102]. 

Chalcones from dietary sources also possess many biological activities. This enables edible plants containing chalcones to be used as therapeutic agents [103]. Tomatoes have been reported to exhibit anticardiovascular, antidiabetic, and anticancer activities [104,105,106]. Anti-inflammatory, antiallergic [107], and antiobesity [108] activities have been reported with naringenin chalcone. It is one of the major bioconstituents of tomatoes [107,108,109]. Other chalcone constituents such as phloretin-3′,5′-di-C-glucoside present in tomatoes have been reported to possess antioxidant properties [96]. Panduratin A, boesenbergin A, and pinostrobin chalcone in tomatoes have been reported for their aphrodisiac properties [110]. 

In a separate description, panduratin A has been reported for its antioxidant, antiobesity, anti-inflammatory, and antimicrobial activities [111,112,113,114]. Even though boesenbergin has been reported to be highly hepatoxic, it has been demonstrated to exhibit anti-inflammatory, antioxidant, and anticancer activities [115]. Protease inhibition, anticancer, and antipyretic activities have been attributed to cardamonin [116]. Antiretroviral activity has been reported for hydroxypanduratin A, pinostrobin, and panduratin chalcone [117,118]. Licochalcone A, a constituent of licorice, has been reported to have a good inhibition of TNF-α, IL-β, and IL-6 inflammatory markers [118,119]. This chalcone along with licochalcone B, C, and D has been associated with antiviral [3], anti-inflammatory [120], antidiabetic [121], antitrypanosomal [122], anticancer [123], and antibacterial [124] activities. Apple containing dihydrochalcone constituents has biological activities against many diseases [125,126]. Phloretin is the most important chalcone present in apple. Phloretin has been demonstrated to possess antioxidant, anticancer, and anti-inflammatory effects [127,128]. As an anticancer agent, it targets the inhibition of GLUT2. It also inhibits the anti-inflammatory markers such as NF-_K_β, TNF-α, etc. [128].

The bioactivities of chalcones obtained from medicinal plants are illustrated in Table 1.

**Figure 6:**
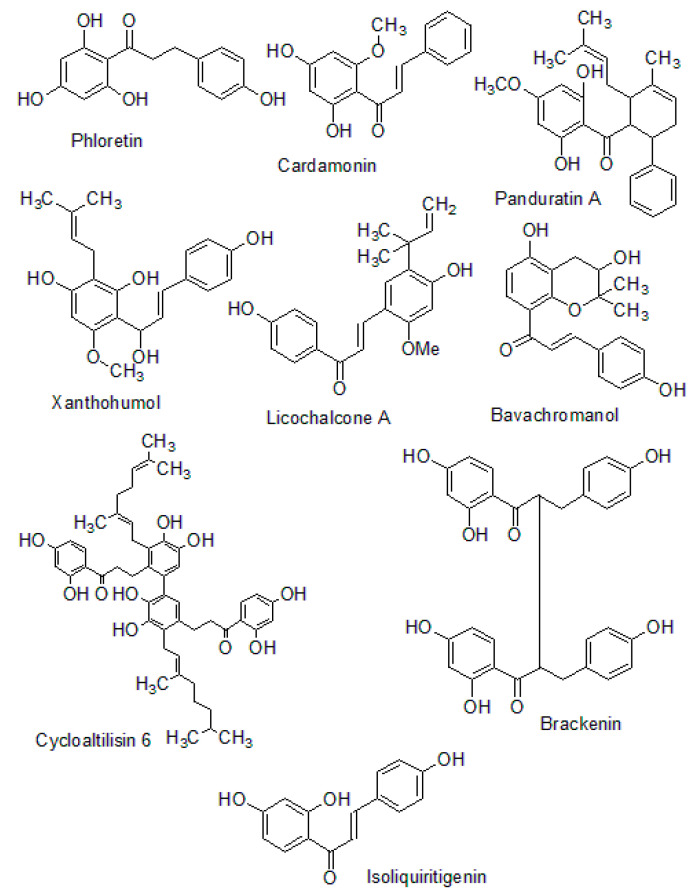
Structures of naturally occurring chalcones.

The structures of naturally occurring chalcones are presented in Figure 6.

## 5. Pharmacokinetics and Toxicities of Chalcones

**Figure 7:**
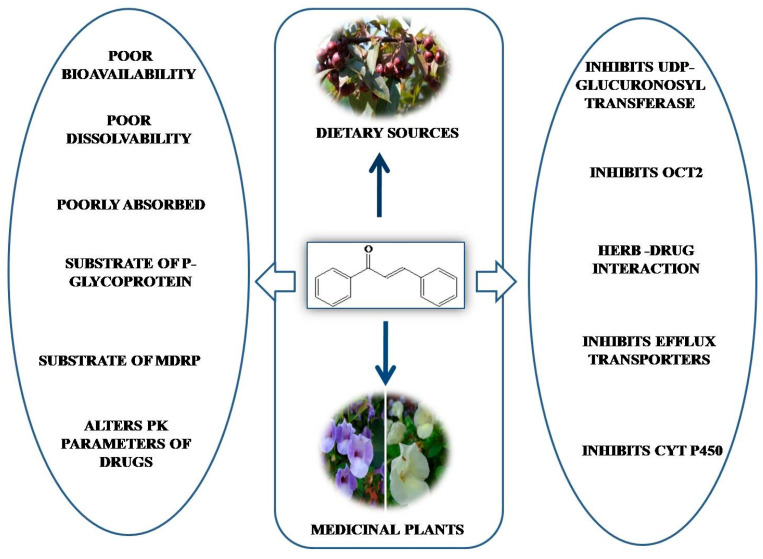
Challenges associated with pharmacokinetics of naturally occurring chalcones.

Although chalcones have a wide range of pharmacological activities, the unavailability of sufficient bioavailability and bioaccessibility data in humans is a major challenge toward their development as therapeutic agents [159]. Synthetic chalcones have been widely studied, whereas the bioavailability of chalcones from natural sources is limited. The expected level of in vivo efficacy in preclinical evaluation has not been reached yet due to poor bioavailability profile. However, optimization of the physiochemical properties of chalcone derivatives could be an important step in their further development as lead molecules or drug candidates. The adsorption, distribution, metabolism, excretion, and toxicity (ADMET) of some naturally occurring chalcones have been studied, but the data do not satisfactorily support their ADMET profile [160,161] (Figure 7).

Studies have shown that amongst many natural chalcones, prenylated derivatives are bioavailable, but they exhibit low bioaccessibility. One such chalcone is xanthohumol obtained in hop plant (*Humulus lupulus*), which upon oral administration by force feeding at extremely higher dosage to rodents (1 g/kg body weight) produces good oral bioavailability, but it does not obtain appreciable accessibility at the site of action. Xanthohumal 4′-O-glucoronide has been found to be the major metabolite in plasma, and unmetabolized xanthohumol has also been detected ten times less concentration after 4 h post administration [162]. In vitro metabolism studies indicate that xanthohumal in human and rat liver microsomes can be freely converted to glucuronides [163]. Gil-Izquierdo et al. (2001) studied the bioavailability of diversely processed juice of *Citrus sinensis* (L.) by mimicking in vitro digestion in stomach as well as the small intestine [164]. They have reported that in mild alkaline medium, 50–60% of the dissolved flavanones (mainly hesperidine) becomes converted to chalcones (hisperidin chalcone). Due to the poor solubility of these chalcones, the bioequivalence is not achieved to the expected level [165,166]. Another chalcone derivative is cardamonin, which is obtained from plants belonging to the Zingiberacea family, which has been reported to be poorly absorbed upon oral administration exhibiting 18% oral bioavailability in mice. It exhibited **a** high volume of distribution, short mean residence, high clearance, and was excreted in bile in its conjugated and unchanged form.

Zhao et al. (2020) studied the pharamacokinetics of phloretin, a naturally occurring dihydrochalcone flavonoid found in apple, pear, roots peels, and juicy fruits peels, by orally administering it to Sprague–Dawley rats. Absorption mechanisms have been investigated in a Caco-2 cell monolayer and by a single pass intestinal perfusion in rats [167]. Phloretin is transported through active transport, efflux protein transport, and by cell bypass. It has been reported to be a substrate of P-glycoprotein (P-gp) and multi-drug resistance protein (MRP2) and found to have low oral bioavailability (8.676%) with colon as the best absorption site.

Naturally occurring chalcones have also been found to affect the pharmacokinetic parameters of drugs when administered simultaneously. Choi et al. (2014) investigated the effect of licochalcone A on the pharmacokinetics of nifedipine and its metabolite dehydronifedipine in rats. Hepatic CYP3A4 metabolizes nifedipine. Oral administration of nifedipine with licochalcone A has been found to inhibit CYP3A4 as well as exhibit the cellular accumulation of rhodamine-123 in MCF-7/ADR cells overexpressing P-gp, leading to a higher peak plasma concentration (Cmaxs) [168]. Boonnop et al. (2017) proposed that the co-administration of *Boesenbergia rotunda* extract with therapeutic drug may cause herb–drug interaction, leading to an alteration of the efficacy and toxicity of the drug. Panduratin A isolated from the *Boesenbergia rotunda* has been reported to cause herb–drug interaction and alter renal cationic drug clearance by inhibiting organic cation transporters (OCT2), which are responsible for the renal excretion of cationic drugs [169].

Recently, Qin et al. (2021) also studied the metabolic and inhibitory effects of isobavachalcone, a natural chalcone obtained from *Psoralea corylifolia*, on efflux transporters, cytochrome P450 and UDP-glucuronosyltransferase enzymes. The glucuronidation of isobavachlacone in the human liver microsome and human intestine microsome has been well characterized with the production of three glucuronides. Moreover, the main contributors for glucuronidation were UGT1 A9, 1A8, 1A7, 1A3, and 1A1. MRP1, MRP4, and BCRP transporters have been found to participate more in glucuronide excretion. Isobavachalcone has been recognized as a broad-spectrum inhibitor against UGT2B7, UGT1A9, UGT1A1, CYP2E1, CYP2D6, CYP2C19, CYP2C9, and CYP2B6 [170]. 

In view of the above facts, to design a chalcone derivative with acceptable ADMET properties, the maximization of its physiochemical properties with modification in the chemical structure would play a crucial role.

## 6. Conclusions and Future Directions

Chalcone scaffolds considered as the key bioactive precursors of plant flavonoids possess huge chemical and biological potential with significance in medicinal chemistry and pharmacology in current times. The chemistry and biological importance of naturally occurring chalcones have not been extensively explored. However, regardless of its versatile medicinal importance, the pharmacokinetics of plant-derived/dietary chalcones is a major challenge. Moreover, there is a lack of preclinical or clinical data on naturally occurring chalcones in the current literature. Further in-depth research studies are required to be carried out to address the pharmacokinetic issues and toxicological aspects related to naturally occurring chalcones and chalcone-derived flavonoids. There are ample scopes for the discovery of lead molecules or drug candidates from naturally occurring bioactive chalcones. Therefore, the proper chemical derivatization of natural chalcones is necessary to obtain novel flavonoid molecules that would play a vital role in the chalcone scaffolds-based discovery of drug molecules.

## Figures and Tables

**Table 1:** Bioactivities of important naturally occurring chalcones.

Sl. No.	Plant Species	Chalcone	Bioactivity	Reference
1	*Dalbergia odorifera* T	Butein (2′,4′,3,4-tetrahydroxychalcone)	Antioxidant activity against lipid and LDL peroxidation	[129]
2	*Humulus lupulus*	Xanthohumol	Antioxidant activity against LDL oxidation	[130]
3	*Broussonetia papyrifera* Vent.	BroussochalconeA	Antioxidant activities due to inhibition of IκBα degradation and iNOS	[131]
4	*Bidens pilosa*	Okanin	Antioxidant activity	[132]
5	*Malotus philippinensis*	11-O-galloylbergenin	Anti-inflammatory activity	[133]
6	*Toussaintia orientalis Verdc.*	2-Hydroxy-3,4,6-trimethoxychalcone	Anti-inflammatory activity against COX-2 enzyme	[134]
7	*Glycyrrhiza inflate*	Licochalcone A	Anti-inflammatory activity	[135]
8	*Humulus lupulus* L.	Xanthohumol B and dihydroxanthohumol	Anti-inflammatory activity by inhibition of production of NO due to the suppression of iNOS	[136]
9	*Psoralea corylifolia*	Isobavachalcone, bavachromene, kanzonol B, 4-hydroxy- lonchocarpin chromenoflavanone	Anti-inflammatory activity due to inhibition of iNOS and COX-2 in LPS-activated microglia; blocks the I-κBα degradation and down-regulated NF-κB level in LPS-stimulated BV-2 microglia	[137,138]
10	*Artocarpus communis*	Arcommunol C, arcommunol D, 5′-geranyl-3,4,2′,4′-tetrahydroxychalcone, prostratol, arcommunol E, 3′-geranyl-3,4,2′,4′-tetrahydroxydihydrochalcone, and 3′-geranyl-3,4,2′,4′-tetrahydroxychalcone	Anti-inflammatory activity by decreased LPS mediated induction of protein expressions of iNOS and COX-2 in RAW 264.7 cells	[139]
11	*Glycyrrhiza inflata*	Licochalcone A and C	Antimicrobial activity by inhibition of NADH-cytochrome *c* reductase	[140]
12	*Boesenbergia rotunda*	Panduratin A	Antimicrobial activity against clinical enterococci	[141]
13	*Angelica keiskei*	Isobavachalcone, bavachalcone broussochalcone	Antibacterial activity against Gram-positive bacteria	[142]
14	*Mallotus philippinensis*	Rottlerin, 4′-hydroxyrottlerin, 1-(5,7-dihydroxy-2,2,6-trimethyl-2H-1-benzopyran-8-yl)-3-phenyl-2-propen-1-one	Antifungal activity	[143]
15	*Maclura tinctoria*	Isobavachalcone	Antifungal activity against Candida albicans and *Cryptococcus neoformans*	[144]
16	*Zuccagnia punctata Cav.*	2′,4′-dihydroxychalcone and 2′,4′-dihydroxy-3′-methoxychalcone	Antifungal activity	[145]
17	*Humulus lupulus*	Xanthohumol	Anti-HIV-1 activity by induction of cytopathic effects, viral p24 antigen and reverse transcriptase in C8166 lymphocytes	[146]
18	*Boesenbergia pandurata*	Hydroxypanduratin A, panduratin A	Anti-HIV-1 protease inhibitory activity	[147]
19	*Glycyrrhiza inflata*	Licochalcone G	Antiviral activity against H1N1 swine influenza	[148]
20	*Erythrina abyssinica*	5-prenylbutein	Anti-plasmodial activity	[149]
21	*Crotalaria orixensis*	Crotaorixin	Antimalarial activity against *Plasmodium falciparum* (Strain NF-54)	[150]
22	*Glycyrrhiza uralensis*	Licochalcone A	Antimalarial activity	[151]
23	*Cyclosorus parasiticus*	Parasiticins C, 2′,4′-dihydroxy-6′-methoxy-3′,5′-dimethylchalcone	Anti-proliferative activity by induction of apoptosis in the HepG2 cell line	[35]
24	*Alpinia* pricei *Hayata*	Flavokawain B	Antiproliferative effect due to induction of G2/M accumulation, autophagy, and apoptosis	[152]
25	*Caesalpinia ferrea Mart*	Pauferrol B, pauferrol C	Inhibitory activities against human topoisomerase II and cell proliferation by induction of apoptosis in human leukemia cells lines (HL 60)	[153]
26	*Boesenbergia rotunda*	Panduratin A	Anti-angiogenic agent	[154]
27	*Angelica keiskei*	4-Hydroxyderricin	Hypotensive and lipid regulatory actions, reduction of serum VLDL levels and hepatic triglyceride	[155]
28	*Artemisia dracunculus* L.	Davidigenin, 2′,4′-dihydroxy-4-methoxydihydrochalcone,4,5-di-*O*-caffeoylquinic acid, 6-demethoxycapillarisin and	Antidiabetic activity as aldose reductase inhibitor	[156]
29	*Lonchocarpus sericeus*	lonchocarpin and derricin	Antiplatelet activity by phosphodiesterase activity inhibition or elevation of intracellular levels cAMP and cGMP or by inhibition of thromboxane formation	[157]
30	*Glycyrrhiza glabra*	Glabrol, 4′-O-methoxy glabridin, hispaglabridin A, glabridin, 4′,7-dihydroxy flavone, 7-hydroxy-4′-methoxy flavone, 3,3′,4,4′-tetrahydroxy-2-methoxychalcone, liquiritigenin, isoliquiritigenin, licuroside, isoliquiritoside and isoononin	Antiobesity and lipid-lowering effects	[158]

## Abbreviations

ADMET: absorption, distribution, metabolism, excretion and toxicity, COX-2: cyclooxygenase-2, CHS: chalcone synthase, cAMP: 3′,5′-cyclic adenosine monophosphate, cGMP: guanosine 3′,5′-cyclic monophosphate, DFR: dihydroflavonol-4-reductase, DMAPP:dimethylallyl pyrophosphate, FLS: Flavonol synthase, F3H: flavanone-3-hydroxylase, GLUT2: glucose transporter 2, I-κBα: IκBα inhibitory-κBα, IFS: isoflavonone synthase, LPS: ipopolysaccharide, MAPKs: mitogen-activated protein kinases, MRP2: multidrug resisteace-associtaed protein 2, NF-κB: nuclear factor-κB, NO: nitric oxide, NOS: nitric oxide synthase, iNOS: inducible NOS, OCT2: organic cation transporter 2, OMTs: O-methyltransferase, PAL: Phenylalanine ammonia-lyase, PTBIB: protein tyrosine phosphatase IB, TNF: tumor necrosis factor, UF3GT: UDP-glucose, flavonoid-3-O-glucosyltransferase.
